# Computer reconstruction of gene networks controlling anxiety levels in humans and laboratory mice

**DOI:** 10.18699/vjgb-25-19

**Published:** 2025-02

**Authors:** E.G. Vergunov, V.A. Savostyanov, A.A. Makarova, E.I. Nikolaeva, A.N. Savostyanov

**Affiliations:** Institute of Cytology and Genetics of the Siberian Branch of the Russian Academy of Sciences, Novosibirsk, Russia Scientific Research Institute of Neurosciences and Medicine, Novosibirsk, Russia Novosibirsk State University, Novosibirsk, Russia; Herzen University, St. Petersburg, Russia; Institute of Cytology and Genetics of the Siberian Branch of the Russian Academy of Sciences, Novosibirsk, Russia; Herzen University, St. Petersburg, Russia; Institute of Cytology and Genetics of the Siberian Branch of the Russian Academy of Sciences, Novosibirsk, Russia Scientific Research Institute of Neurosciences and Medicine, Novosibirsk, Russia Novosibirsk State University, Novosibirsk, Russia

**Keywords:** differentially expressed genes, cingulate cortex, automatic text analysis, scientific publications, computer reconstruction, gene networks, mouse model with high-normal-low anxiety behavior, дифференциально экспрессирующиеся гены, поясная кора головного мозга, автоматический анализ текстов, научные публикации, компьютерная реконструкция, генные сети, модель мышей с поведением высокой-нормальной-низкой тревожности

## Abstract

Anxiety is a normotypic human condition, and like any other emotion has an adaptive value. But excessively high or low anxiety has negative consequences for adaptation, which primarily determines the importance of studying these two extreme conditions. At the same time, it is known that the perception of aversive stimuli associated with anxiety leads to changes in the activity of the brain’s cingulate cortex. The advantage of animals as models in studying the genetic bases of anxiety in humans is in the ability to subtly control the external conditions of formation of a certain state, the availability of brain tissues, and the ability to create and study transgenic models, including through the use of differentially expressed genes of small laboratory animals from the family Muridae with low and high anxiety. Within the framework of the translational approach, a three-domain potential gene network, which is associated with generalized anxiety in humans, was reconstructed using mouse models with different levels of anxiety by automatically analyzing the texts of scientific articles. One domain is associated with reduced anxiety in humans, the second with increased anxiety, and the third is a dispatcher who activates one of the two domains depending on the status of the organism (genetic, epigenetic, physiological). Stages of work: (I) A list of genes expressed in the cingulate cortex of the wild type CD-1 mouse line from the NCBI GEO database (experiment GSE29014). Using the tools of this database, differences in gene expression levels were revealed in groups of mice with low and high (relatively normal) anxiety. (II) Search for orthologs of DEG in humans and mice associated with anxiety in the OMA Orthology database. (III) Computer reconstruction using the ANDSystem cognitive system based on (a) human orthologous genes from stage (III), (b) human genes from the MalaCards database associated with human anxiety. The proven methods of the translational approach for the reconstruction of gene networks for behavior regulation can be used to identify molecular genetic markers of human personality traits, propensity to psychopathology.

## Abstract (ru)

Тревожность – это нормотипичное состояние человека, которое, как и любая другая эмоция, имеет адаптивное значение. Но состояние чрезмерно высокой или низкой тревожности влечет за собой негативные последствия для адаптации, что в первую очередь обусловливает важность изучения этих двух крайних состояний. При этом известно, что в условиях восприятия аверсивных стимулов, ассоциированных с тревожностью, изменяется активность поясной коры мозга. Преимущество животных как моделей при изучении генетических оснований тревожности у человека связано с возможностью тонко контролировать внешние условия формирования определенного состояния, доступностью тканей мозга и возможностью создавать и изучать трансгенные модели, в том числе с использованием дифференциально экспрессирующихся генов мелких лабораторных животных из семейства мышиных с низкой и высокой тревожностью. В рамках трансляционного подхода была реконструирована трехдоменная потенциальная генная сеть, которая ассоциирована с генерализованной тревожностью у человека, по моделям мышей с разным уровнем тревожности путем автоматического анализа текстов научных статей. Один домен ассоциирован с пониженной тревожностью у человека, второй – с повышенной, третий служит диспетчером, который активирует один из двух доменов в зависимости от статуса организма (генетического, эпигенетического, физиологического). Этапы работы: (I) из базы данных NCBI GEO взят список генов, экспрессирующихся в поясной коре головного мозга линии мышей дикого типа CD-1 (эксперимент GSE29014). С помощью инструментов этой базы выявлены различия в уровнях экспрессии генов в группах мышей с низкой и высокой (относительно нормальной) тревожностью; (II) поиск ортологов ДЭГ у человека и мышей, ассоциированных с тревожностью в базе данных OMA Orthology; (III) компьютерная реконструкция с помощью когнитивной системы ANDSystem на основе генов-ортологов человека из этапа (II), генов человека из базы данных MalaCards, ассоциированных с тревожностью человека. Апробированные методы трансляционного подхода для реконструкции генных сетей регуляции поведения могут использоваться для выявления молекулярно-генетических маркеров черт личности человека, склонности к психопатологии

## Introduction

Anxiety is a normotypical human condition (Malezieux et al., 2023), and like any other emotion has adaptive value (Stein, Bouwer, 1997). However, a state of excessive anxiety or the complete absence of it has negative consequences for adaptation (Penninx et al., 2021). It is considered proven (Hettema et al., 2001) that a combination of genetic and environmental factors is the cause of extreme variations in the expression of anxiety

The advantage of animals as models in studying the genetic basis of anxiety in humans is due to the ability to precisely control the external conditions for the formation of a particular state, because of the availability of brain tissue, and the ability to create and study transgenic models (Vandamme, 2014; Chadaeva et al., 2023; Krause et al., 2023), including using differentially expressed genes (DEGs) of small laboratory animals from the low anxiety behavior (LAB) and high anxiety behavior (HAB) mouse families (Gryksa et al., 2023). When comparing animal and human models, genetic studies of humans with generalized anxiety disorder are compared with rodent models obtained by exposure to stressful stimuli (Koskinen, Hovatta, 2023). The relevance of such models for understanding the molecular basis of anxiety is evident

Currently, a large number of genes are considered in explaining anxiety in humans (Otowa et al., 2016; Koskinen, Hovatta, 2023; Mucha et al., 2023). The molecular mechanisms of anxiety in both humans and animals are related to differential gene activity of neurotransmitter systems, predominantly serotonin and dopamine, as well as the involvement of other monoamines and GABA (Morris-Rosendahl, 2002; Nuss, 2015; Gottschalk, Domschke, 2017; Galyamina et al., 2018; Moraes et al., 2024; Strom et al., 2024). At the same time, the role of genetic polymorphisms in determining the level of anxiety has been noted (Sen et al., 2004; Ivanov et al., 2019).

Genetic markers of anxiety in mice and humans are similar in many ways, which allows the results obtained in animals to be transferred to understanding the mechanisms of anxiety in humans (Hovatta, Barlow, 2008; Hettema et al., 2011; Brasher et al., 2023). It has been pointed out that the manifestation of genetic polymorphisms is highly modified by sociocultural factors and, in general, the relationship between anxiety and genotype in humans is significantly modulated by environmental conditions (Schinka et al., 2004; Ebstein, 2006; Meng et al., 2024; Petrican et al., 2024).

Attempts to identify genetic markers of behavioral traits based on the analysis of candidate genes are usually ineffective due to the fact that there are no single genes that unambiguously determine behavior (Duncan et al., 2014; Bruzzone et al., 2024). This is explained by the fact that the formation of organisms’ phenotypic characteristics is controlled not by individual genes, but by gene networks – groups of coordinately functioning genes interacting with each other through their products – RNA, proteins, and metabolites (Kolchanov et al., 2000, 2013). It is gene networks, functioning on the basis of information encoded in genomes, that ensure the formation of all phenotypic traits of organisms (molecular, biochemical, cellular, physiological, morphological, etc.) (Kolchanov et al., 2013).

We believe that the reconstruction and analysis of gene networks are promising approaches to understanding the molecular genetic mechanisms underlying the formation of human personality characteristics, including those that, like anxiety, are induced by environmental factors. Reconstruction of gene networks and their functional modules is based on molecular genetic data presented in scientific publications and factographic databases, such as human and animal genome sequencing data, information on differentially expressed genes, allelic polymorphisms associated with target phenotypic characteristics of organisms, and others (Mostafavi et al., 2008; Krämer et al., 2014; Szklarczyk et al., 2015; Chen et al., 2016; Ivanisenko et al., 2022).

However, the reconstruction of human anxiety gene networks cannot be performed on the basis of in vivo experimental studies that would require sampling of biological brain tissues to obtain molecular genetic data. Therefore, in this work, a translational approach was used based on the analysis of data obtained by L. Czibere et al. (2011) in experiments on mice, in which they studied the differential expression of genes (DEGs) in the cingulate cortex of a line of wild-type CD-1 mice with different levels of anxiety

This experiment showed that mice with high anxiety exhibited a more passive coping strategy than mice with low anxiety, which is reminiscent of the clinical comorbidity of anxiety and depression (their co-occurrence) observed in psychiatric patients (Czibere et al., 2011). This was the rationale for using mouse DEG data to reconstruct human gene networks involved in the control of different levels of anxiety. The details of this translational approach are described below.

In the reconstructed potential human gene network, we identified three functional domains, one of which is responsible for the reaction of reduced anxiety, another domain is responsible for the reaction of increased anxiety, and the third plays the role of a dispatcher that activates one of the other two domains depending on the genetic, epigenetic, physiological status of the organism and environmental conditions

## Materials and methods

Experimental data. In this study, we used the data from the work of L. Czibere et al. (2011), in which 25 specimens of wild-type mice of the CD-1 line of one generation (Mus musculus Linnaeus, 1758; https://www.ncbi.nlm.nih.gov/ Taxonomy/Browser/wwwtax.cgi?mode=Info&id=10090) were subjected to stress exposure (swimming in cold water for 10 min). Afterward, using the MouseWG-6 v1.1 Expression BeadChip-system expression chip (46,132 samples), L. Czibere et al. (2011) assessed gene expression levels in the cingulate cortex of these mice. Experimental animals were divided on the basis of behavioral tests in the sleeves of an elevated cross-shaped maze into three groups: with low (low anxiety behavior, LAB), normal (normal anxiety behavior, NAB) and high (high anxiety behavior, HAB) anxiety (Czibere et al., 2011). The results of the experiment are presented in the NCBI GEO database with the index GSE29014 (https:// www.ncbi.nlm.nih.gov/geo/query/acc.cgi?acc=GSE29014).

Computer analysis methods. The list of genes expressed, according to (Czibere et al., 2011), in the cingulate cortex of mice (experiment GSE29014) was taken from the NCBI GEO database (https://www.ncbi.nlm.nih.gov/geo/query/acc. cgi?acc=GSE29014). Identification of differences in gene expression levels between groups of mice with different levels of anxiety was performed using the NCBI GEO toolkit (https://www.ncbi.nlm.nih.gov/geo/geo2r/?acc=GSE29014). The OMA Orthology database (https://omabrowser.org/oma/ home/) was used to search for orthologs of DEGs in humans and mice associated with anxiety.

Reconstruction of potential human gene networks associated with high- and low-level generalized anxiety states was performed on the basis of human genes orthologous to mouse genes differentially expressed in the cingulate cortex. For this purpose, we used ANDSystem, a cognitive system developed at the Institute of Cytology and Genetics SB RAS (Ivanisenko et al., 2019), which uses machine reading and artificial intelligence methods to automatically extract knowledge and facts from large genetic data sources – texts of tens of millions of scientific articles and patents and thousands of factographic databases. The ANDSystem knowledge base currently contains information on 2 million genes and proteins, 46 thousand diseases, tens of thousands metabolites and biological processes, and tens of millions intermolecular interactions (Ivanisenko et al., 2024).

## Results

**Fig. 1.**
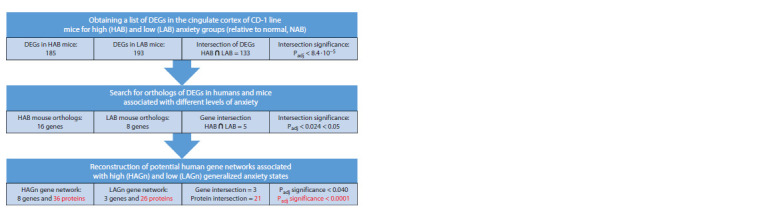
Basic steps in reconstructing a potential human gene network associated with high and low levels of anxiety.

The basic framework for data analysis, starting with the generation of a list of DEGs in the cingulate cortex of mouse line CD-1 for high (HAB) and low (LAB) anxiety groups, which includes a search for orthologs of differentially expressed genes in humans and mice associated with anxiety, and culminating in the reconstruction of potential human gene networks associated with anxiety levels, is shown in Figure 1. Let us review the main results of this approach.

Obtaining a list of DEGs in the cingulate cortex of CD-1 line mice for HAB and LAB anxiety groups

First of all, we searched for differentially expressed genes in the cingulate cortex of CD-1 mice that distinguish (a) the high anxiety group (HAB) from the normal anxiety group (NAB) and (b) the low anxiety group (LAB) from the normal anxiety group (NAB). When comparing the HAB and NAB groups, 185 DEGs were identified, and when comparing the LAB and NAB groups, 193 DEGs were identified (Fig. 1). The number of total DEGs in mice identified in the HAB/NAB and LAB/NAB comparisons is 133. Assessing the significance of such a strong overlap using a Bonferroni-corrected hypergeometric distribution for multiple comparisons yields a Padj < 8.4·10–5 (Fig. 1).

It can be hypothesized that the stress responses of the two compared pairs of groups of mice corresponding to increased or decreased anxiety are parts of some large gene network that determines the level of anxiety in the stress response.

Search for orthologs of DEGs in humans and mice associated with anxiety

Identification of human genes orthologous to mouse DEGs, identified by comparing gene networks responsible for differences in anxiety levels between the LAB/NAB and HAB/NAB groups of mice, was performed using the OMA Orthology database (https://omabrowser.org/oma/ home/). For this purpose, a Python script was written that compared mouse ID genes with human orthologs and produced IDs for human genes. In total, such comparisons identified 8 human orthologous genes based on DEGs for LAB/NAB mice and 16 based on DEGs for HAB/NAB mice. The number of human orthologous genes common to the two lists is 5. Assessing the significance of the overlap using a Bonferronicorrected hypergeometric distribution for multiple comparisons yields a value of Padj <0.024 < 0.05 (Fig. 1).

Reconstruction of potential human gene networks associated with high- and low-level generalized anxiety states

This task was solved using the cognitive system ANDSystem. Two types of data were used. First of all, a sample containing the previously identified 19 different human orthologous genes. And, in addition, 176 human genes associated with anxiety and depressive spectrum disorders, which were extracted from the MalaCards database (https://www.malacards. org/card/anxiety#Genes).

On this basis, two potential human gene networks associated with (a) high anxiety (HAGn, High Anxiety Gene Network, containing 8 genes, 36 proteins), and (b) low anxiety (LAGn, Low Anxiety Gene Network, containing 3 genes, 26 proteins) were reconstructed using ANDSystem

**Fig. 2.**
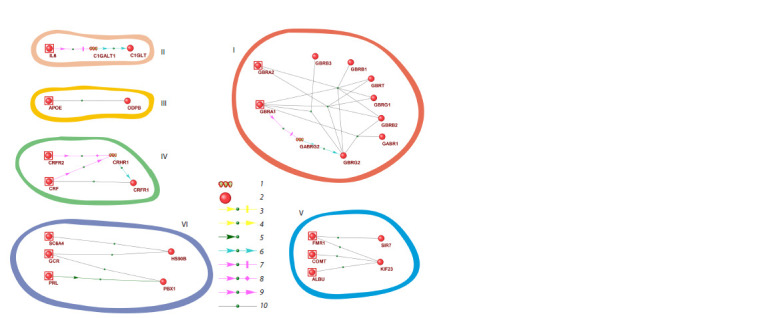
Visualization of the potential human LAGn gene network responsible for low anxiety state in humans. Roman numerals indicate isolated clusters. Arabic numerals denote: 1 – gene, 2 – protein, 3 – suppression of protein activity, 4 – enhancement of protein activity, 5 – catalytic reaction, 6 – expression, 7 – suppression of gene expression, 8 – regulation of gene expression, 9 – enhancement of gene expression, 10 – protein-protein interaction.

The LAGn gene network responsible for the state of low anxiety level contains a large cluster of 10 interacting proteins and genes and 5 isolated small-sized clusters (Fig. 2).

**Fig. 3.**
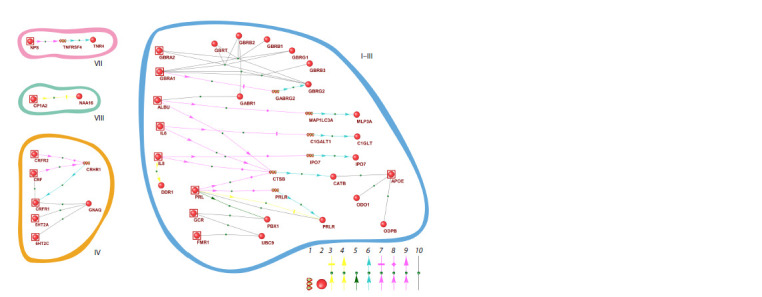
Visualization of the potential human HAGn gene network responsible for the state of high anxiety in humans The labeling is analogous to that presented in Figure 2.

In the HAGn gene network responsible for the state of high anxiety, first, a large cluster of 32 interacting proteins and genes is distinguished, followed by a medium-sized cluster of 7 proteins and genes, as well as 2 isolated smaller clusters (Fig. 3).

Note that the large HAGn cluster (Fig. 3, I–III) includes the entire three LAGn clusters (Fig. 2, I–III), and the mediumsized HAGn cluster (Fig. 3, IV) includes the entire LAGn cluster (Fig. 2, IV). Two HAGn clusters (Fig. 3, VII, VIII) and two LAGn clusters (Fig. 2, V, VI) have no counterparts among clusters of the other gene network. Although clusters IV, VII, VIII may have overlapping proteins with other clusters in the other gene network, even then their roles in linkages in “their” clusters are different from their roles in clusters in the other gene network.

Both networks (LAGn and HAGn) have 3 genes in common. Assessment of the significance of such intersection of LAGn and HAGn according to the hypergeometric distribution with Bonferroni correction for multiple comparisons gives Padj <0.040 (< 0.050) (Fig. 1). Both networks (LAGn and HAGn) share 21 common proteins. Assessment of the significance of such intersection of LAGn and HAGn according to the hypergeometric distribution with Bonferroni correction for multiple comparisons gives Padj < 0.0001 (Fig. 1).

## Discussion

**Fig. 4.**
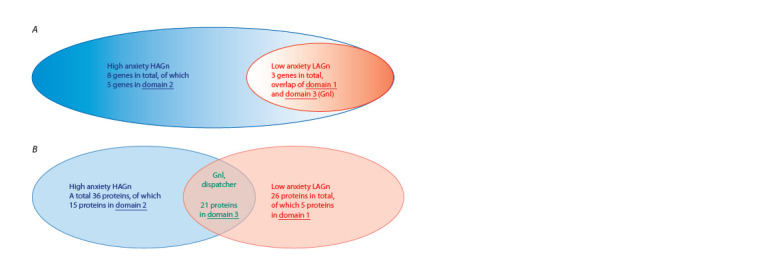
Qualitative scheme of interaction between LAGn (gene network of reduced anxiety), HAGn (gene network of increased anxiety) and GnI (dispatcher that activates domain 1 or domain 2). A – distribution of genes, B – distribution of proteins encoded by the genes.

It can be assumed that the 3 identified genes common to the two networks (LAGn and HAGn) form a special gene network – GnI (Gene Network Interface), which regulates the interaction between the gene networks LAGn and HAGn, which are responsible for the formation of low and high anxiety states. Figure 4 shows a qualitative schematic of the interaction between LAGn, HAGn and GnI:

interaction between LAGn, HAGn and GnI: • Domain 1 (part of LAGn) is responsible for the low anxiety response; • Domain 2 (part of HAGn) is responsible for the higher anxiety response; • Domain 3 (GnI, the common part for both LAGn and HAGn) acts as an interface between domains 1 and 2. It plays the role of a dispatcher that activates domain 1 or domain 2 depending on the genetic, epigenetic, physiological status of the organism. A discussion of the approach based on the existence of such a dispatcher is given in (Shin et al., 2024).

As our DEG analysis based on the GSE29014 experi- ment shows, a similar three-domain structure is evident for the interactions between two sets of genes associated with low (LAB) and high (HAB) anxiety in mice, as well as ortholo- gous genes (Fig. 1). As Figure 4 shows, the interactions of LAGn, HAGn, and GnI are complex and need further dedicated study

We chose the cingulate cortex in our work to identify genes, the expression of which after stress response is associated with an increase or decrease in the level of anxiety in experiObtaining mental mice, because fMRI studies (de la Pena-Arteaga et al., 2024) had shown altered activity of the cingulate cortex under conditions of perception of aversive stimuli associated with anxiety.

Let us draw attention to the fact that the experiments conducted by L. Czibere et al. (2011) on a genetic line of wild-type CD-1 mice revealed two opposite reactions to the same stressor. This can be explained by the presence of latent genomic variability in the population of the examined mice (the presence of a spectrum of polymorphisms or epigenetic modifications affecting a variety of genomic loci). Perhaps this may explain the fact that the degree of anxiety is a continuum, the scores of which continuously vary from low through medium to high values (Friligkou et al., 2024).

Our analysis showed that the qualitative differences in mice between low (LAB) and normal (NAB) levels of anxiety on the one hand, and high (HAB) and normal (NAB) on the other hand, revealed in the experiment of L. Czibere et al. (2011), may lie in gene networks functioning in the cingulate cortex that provide contrasting states of anxiety relative to normal.

Earlier genetic studies have shown the association of anxiety with genes for brain monoamine systems (Lesch et al., 1996; Murphy et al., 2013). Polymorphisms in serotonin system genes, including genes encoding serotonin receptors and serotonin transporters, are associated with different levels of anxiety (Purves et al., 2020).

The set of human genes we have identified as part of the reconstructed potential gene networks includes genes of monoamine brain systems. These include, for example, serotonin receptors 5HT2A and 5HT2C (a potential gene network domain for high anxiety states). These receptors belong to the G-protein-coupled receptor (GPCR) superfamily and, through interaction with GPCRs, transmit extracellular signals to the interior of cells. The receptors mediate the effects of a large number of compounds affecting depression, schizophrenia, anxiety, hallucinations, dysthymia, sleep patterns, eating behavior, and neuroendocrine functions (Van Oekelen et al., 2003). This is in good agreement with the monoamine hypothesis of anxiety (Morris-Rosendahl, 2002; Gottschalk, Domschke, 2017; Hirai et al., 2024).

Our reconstructed potential human gene networks also include interactions with genes encoding proteins such as COMT or APoE, which are not related to neurotransmitters but are associated with anxiety and depression through involvement in the regulation of a wide range of metabolic processes (Koskinen, Hovatta, 2023).

It has been established (Gunthert et al., 2007) that there is a functional relationship between genetic polymorphisms and anxiety levels for groups of people living in different environmental conditions. Environmental factors have been shown to interact with genetic markers of anxiety in complex ways, in some cases leading to inversion of allelic polymorphism effects when living conditions change (Schinka et al., 2004; Sen et al., 2004; Ivanov et al., 2019; Meng et al., 2024; Petrican et al., 2024).

It can be hypothesized that the level and directionality of anxiety as a stress response depends on: (a) genes directly involved in neural signal processing; (b) genes regulating other body functions (metabolic, physiological…); (c) the presence of hidden genomic variability – epigenetic modifications, polymorphisms, etc. in the above two groups of genes (a) and (b).

It is known that the results obtained on animal models in drug development cannot always be adequately extrapolated to humans (Hackam, Redelmeier, 2006). There may also be a concern that a study on 25 individuals from a single generaFig tion of a wild mouse line may lead to simplistic conclusions and limited understanding of the complex network of genes involved in anxiety, and any errors or inaccuracies in the original data may lead to incorrect conclusions about the role of genes in anxiety.

In our approbation of the translational approach, such issues were addressed as follows: human and mouse orthologous genes obtained from a list of mouse cingulate cortex DEGs were matched to a set from the MalaCards database (176 human genes that are associated with generalized anxiety and anxiety and depressive spectrum disorders for humans). The MalaCards database provides a set of references to papers describing relevant experiments, allowing validation for each case. After such a comparison, the reconstruction of potential (i. e. assuming special further study) gene networks for humans was carried out with the help of the ANDSystem cognitive system on the basis of automatic analysis (and resolution of inaccuracies and contradictions found in them) of 6 million texts of articles from leading publications on biological topics. Thus, the impact of inaccuracy or insufficiency of the original data in the LAB and NAB mouse groups was reduced to a negligible level in our approbation

## Conclusion

Based on the software resources used in our work and the generated algorithm for analyzing differential expression of genes data, we developed a software module for computer reconstruction of gene networks involved in the regulation of stress response leading to anxiety of different levels.

Within the framework of the translational approach, a three-domain potential gene network, which is associated with generalized anxiety in humans, was reconstructed using mouse models with different levels of anxiety by automatically analyzing the texts of scientific articles. One domain is associated with reduced anxiety in humans, the second with increased anxiety, and the third is a dispatcher who activates one of the two domains depending on the status of the organism (genetic, epigenetic, physiological).

We believe that this approach can be modified to reconstruct gene networks controlling anxiety and other behavioral reactions in stress responses of other types.

Limitations of the present study

The human multidomain gene network we reconstructed, which is associated with generalized anxiety, is potential, that is, it implies dedicated further study and refinement. Thus, this paper takes an initial step in investigating the domains of the gene network that is associated with human anxiety

## Conflict of interest

The authors declare no conflict of interest.
